# Employment status, psychological needs, and mental health: Meta-analytic findings concerning the latent deprivation model

**DOI:** 10.3389/fpsyg.2023.1017358

**Published:** 2023-03-02

**Authors:** Karsten Ingmar Paul, Hannah Scholl, Klaus Moser, Andrea Zechmann, Bernad Batinic

**Affiliations:** ^1^Chair of Business and Social Psychology, Friedrich-Alexander-Universität Erlangen-Nürnberg, Nürnberg, Germany; ^2^Institute of Psychology, Johannes Kepler University Linz, Linz, Austria

**Keywords:** unemployment, mental health, latent functions, time structure, social contact, status, collective purpose, activity

## Abstract

Marie Jahoda’s latent deprivation model proposes that unemployed people have a worse mental health compared to employed people. This is because they suffer not only from a lack of the manifest function of employment (earning money), but also from a lack of five so-called latent functions of employment: Time structure, social contact, collective purpose (i.e., the sense of being useful to other people), status, and activity. In order to test the basic assumptions of this theory, a study based on meta-analytic methods was conducted. Results showed that employed people reported higher levels on all five latent functions, as well as on the manifest function, compared to unemployed people. They also report more latent functions than people who are out of the labor force (OLF). Moreover, OLF-people reported more manifest and latent functions than unemployed people. Specific analyses for three OLF-subgroups found retired people to be almost as deprived of the latent functions (but not the manifest function) as unemployed people, while students were more similar to employed people but still experienced some manifest and latent deprivation. For homemakers, the effect sizes pointed in the expected direction, but they were not significant. Thus, the proposition that employment is the best provider of the latent functions was generally endorsed, although homemakers need further scrutiny in future studies. All latent functions, as well as the manifest function, emerged as significant independent predictors of mental health, when the influence of the other manifest and latent functions was controlled. Together, the dimensions in the model explained 19% of variation in mental health.

## Introduction

The question of whether employment, i.e., working for pay, tends to have more positive or more negative effects on well-being and mental health is probably one of the most important issues in the scientific study of modern economies. First, it is important for theoretical reasons, for example with regard to labor market models assuming that working time is generally less desirable than leisure time and must thus be financially compensated (Franz, 2013). However, if employment turns out to be a source of well-being, financial compensation is only one of several reasons why people aim to work. Second, it is relevant for public health. For example, if employment promotes and unemployment impairs mental health, the costs of treatment of mental health problems of millions of unemployed people will be considerable (Paul and Moser, 2009). Third, it is also relevant for specific policy decisions, e.g., on the effects of the introduction of an unconditional basic income, because the topic pertains to the question of whether people will still opt for employment when the financial need to do so is greatly diminished. Finally, from a psychological and maybe even from a sense-seeking perspective, the question whether employment is primarily a source of pleasure or pain is also relevant for every person as an individual who must decide how to spend the limited time on earth, and what, for example, we should expect from and strive for in the world of work. Yet, despite its importance, the question has received only limited theoretical or empirical research attention.

The most frequently cited theory pertaining to this topic is probably Jahoda’s (Jahoda, 1981, 1982, 1997) latent deprivation model. As far as we know, it is the only theoretical psychological model that was specifically created to answer the questions whether employment is generally beneficial for mental health and why that may be the case. The model proposes that employment does not only provide a manifest function, i.e., a financial income assuring an employee’s physical survival. It also provides five so-called latent functions, which are “unintended consequences” (Merton, 1957) of paid work that are nevertheless important for the maintenance of mental health. These are time structure, social contact, status, collective purpose, and activity.

While Jahoda’s theory has been tested in numerous empirical investigations, the results vary widely, leading to some uncertainty concerning its validity. In particular, it is still unclear (a) whether employment is really the best provider of the latent functions, while all other possible sources such as education, family life, or leisure can only provide limited amounts; and (b) whether the fulfillment of the manifest and latent functions indeed explains a sizable part of the variation in people’s mental health. Aiming to answer these questions, the present paper reports the first published meta-analysis on the latent deprivation model.

### The latent deprivation model

In her renowned Marienthal-study (Jahoda et al., 1933/1975), Jahoda painted a devastating portrait of the psychological state of the inhabitants of an Austrian village who had become unemployed after the only large local employer – a textile factory – was shut down during the Great Depression. Later, she went on to formulate a theoretical model integrating her main findings from Marienthal and from her other studies on unemployment (e.g., Jahoda, 2019) in order to explain the psychological mechanisms behind the harmful effects of joblessness.

Based on Merton’s (1957) distinction between manifest, i.e., obvious and intended, and latent, i.e., hidden and unintended, functions of social institutions, Jahoda (1981, 1982, 1997) specified six functions of employment. One, earning a living, was described as the *manifest function*: When people are asked why they hold down a job, their first answer usually is that they have to earn money in order to sustain themselves and their families. In addition, Jahoda (1981, 1982, 1997) also specified five latent functions of employment: (1) *Time structure*: From early childhood on, people in modern societies are enrolled in institutions that enforce a strict structure of the day, the week and the year, such as a kindergarten, school, university, and later employment. Thus, people become so deeply accustomed to externally imposed rhythms of time that they are not able to structure their time for themselves when, through job loss, the externally imposed time structure breaks down. The huge amount of time that is suddenly available for personal discretion becomes a “tragic gift,” leading to chaotic and squandered days, which in turn lead to negative emotions and a decline in mental health (Jahoda et al., 1933/1975, p.83). (2) *Social Contact*: Almost any kind of job provides contact with a range of other people on a regular basis, which according to the latent deprivation model (Jahoda, 1981, 1982, 1997) is important for maintaining a good mental health. Jahoda notes that these social contacts are not only important for single people, but also for people with a family, because they broaden one’s social horizon beyond the narrow confines of family relationships. (3) *Collective Purpose* pertains to the feeling of being useful to others and leading a meaningful life. This latent function is about the perception that one is part of collective projects that transcend one’s restricted individualistic existence and give it a deeper meaning. Jahoda assumes that practically every form of employment allows the respective job incumbent to construe some form of collective purpose around the job, which is beneficial for well-being. (4) *Status* refers to experiences of being valued and appreciated by the people around a person and by society at large. According to the latent deprivation model, being employed and earning one’s own money itself confers a certain amount of status and just about every job is more respected than being unemployed. Since appreciation is highly important for the self, experiencing a certain status positively influences mental health. (5) *Activity:* While Jahoda assumed that being active had positive effects on psychological well-being, she also assumed that humans tend to be idle if they are not challenged by externally set goals that push them into activity. Employment regularly sets such goals and thus improves psychological well-being.

Jahoda conceptualized all five latent functions as corresponding with psychological needs. In line with contemporary conceptualizations (e.g., Deci and Ryan, 2000), Jahoda (1981, 1982, 1997) assumed these needs to be required psychological inputs. In other words: Satiation of these needs is necessary in order to maintain psychological well-being. A lack (i.e., deprivation) of these inputs will lead to psychological suffering.

While she did not explicitly specify whether she assumed the needs for social contact, status, meaning etc., to be innate or learned, her writing shows that she assumed them to be present in pre-modern societies too, suggesting a high level of ubiquitousness across different times and cultures. And she made it clear (for example by calling these needs “deep seated”) that she saw them as important elements of every human’s psychological constitution.

Furthermore, the latent deprivation model assumes employment to be the only source of latent functions that is able to provide them in a sufficient amount and with sufficient regularity in order to ensure continued psychological well-being. Other sources of the latent functions do exist – for example, active leisure/hobbies or volunteer work – but they are usually not able to act as a complete substitute for employment. As a result, people who are out of employment, particularly unemployed people, will frequently experience symptoms of psychological distress due to the latent functions not being fulfilled.

### Unresolved questions

The latent deprivation model has been tested in several empirical studies, with the results frequently endorsing its assumptions. However, some important questions remain unresolved.

#### Latent functions of employment or specific characteristics of unemployment?

Jahoda assumed that employment is the main provider of the manifest and latent functions in contemporary societies, while other sources such as leisure or religion should play a far less prominent role. As a consequence, job loss inevitably leads to a deprivation, because unemployed people will not be able to find an adequate substitute for employment as a source of the manifest and latent functions. Thus, Jahoda interpreted the deprivation of time structure, social contact etc., that unemployed people experience to be a direct result of their lack of paid work.

An alternative theoretical possibility could be that low scores on time structure, social contact etc., are specific characteristics of unemployment. Unemployment might be a life situation that is very different from other forms of non-employment, such as retirement, homemaking, or going to university and is distinctly characterized by low levels of Jahoda’s six dimensions. For example, low self-perceived status might be a sign of a stigma that is specifically attached to unemployed people, but is not attached to other people who are out of employment such as pensioners (Paul and Batinic, 2010).

In order to clarify this aspect of the latent deprivation model, it is necessary to compare employed people not only to unemployed people, but also to other groups of people who are out of employment, but who are not unemployed. This group, usually called “out-of-labor-force” (OLF) consists of people who do not actively participate in the labor market such as students, homemakers, and retirees (Rengers, 2004). The label “main provider of manifest and latent functions” for employment would only be appropriate, if there are significant differences between employed people and *all* non-employed people, not only unemployed persons, but also OLF-persons.

Labor statisticians usually divide a country’s adult population into three groups: (1) the employed, (2) the unemployed, who together form the labor force, and (3) people currently out of the labor force. People out of the labor force (OLF) are all individuals that are (a) intentionally not employed though they could be part of the work force, i.e., students in higher education, homemakers, and retirees. Note, that there are also OLF-individuals that are (b) unintentionally not employed, in particular those that are not able to work (due to, for example, a severe health impairment). The category also includes the so-called “discouraged workers,” i.e., individuals who would accept a job if offered one, but have given up to actively search for work. We do not include these two groups because it is highly probable that they are in a state of at least partially unfulfilled needs, or, more generally, incongruency, which in turn is related to an impairment of mental health (Paul and Moser, 2006).

Results from empirical studies on this problem are equivocal. While comparisons between employed and unemployed people regularly resulted in clear differences for all latent functions, comparisons between employed people and OLF people often painted an unclear picture, with some of the latent functions showing no difference between these groups (e.g.,. Henwood and Miles, 1987; Evans and Banks, 1992). This casts some doubt on Jahoda’s conceptualization of the latent functions as being mainly provided by employment.

Research question 1: Do employed people report a higher level of fulfillment of latent functions not only in comparison to unemployed people, but also in comparison to OLF people (students, homemakers, and retirees)?

#### Do the latent and manifest functions of employment predict mental health?

Conceptually, Jahoda describes the latent functions of employment as necessary experiences reflecting basic psychological needs that must be satiated regularly and to a sufficient degree in order to maintain mental health. Thus, each of the latent functions should predict mental health and the combined set of the latent and manifest functions should explain a substantial amount of variance in mental health.

There exist several studies where one or more of the latent functions were not significantly associated to mental health [e.g., time structure and collective purpose in Isaksson (1989) or time structure in Creed and Watson (2003)]. Typically, these were small sample sized studies with low power, though. Using the considerably more powerful method of meta-analysis and extending the study base, we expect to find clearly significant bivariate associations for each of the latent and manifest functions and thus we can answer the question whether all six dimensions are related to mental health.

All variables in the model are typically associated with each other, though to a varying degree (e.g., Creed and Macintyre, 2001; Paul and Batinic, 2010). That leads to the question of whether one or more of the latent and manifest functions might be redundant, when the whole set of variables is used simultaneously in order to predict mental health. For example, collective purpose is about the experience of meaning and purpose in life by cooperating with others in order to achieve a collective goal. This clearly implies a minimum level of cooperation with others, linking the concept to social contact. It also implies a certain level of activity, linking it with another latent function. In fact, with regard to extant multivariate analyses, results were inconclusive. Multiple regression analyses predicting mental health with all manifest and latent functions simultaneously usually resulted in only a subset of the predictors becoming significant. However, which of the latent functions were significant and which were insignificant differed between analyses (e.g., Creed and Macintyre, 2001; Muller et al., 2004; Paul and Batinic, 2010; Selenko et al., 2011). One explanation of this inconsistent pattern of findings might be the comparatively low sample size in most of these analyses, resulting in a low stability of the intercorrelation matrix the multiple regression analyses were based on. Since meta-analytic methods will lead to much more stable correlations, a multiple regression based on such a meta-analytically derived correlation matrix will help to clarify both questions, i. e., whether fulfillment of all functions is related to mental health and whether they incrementally contribute to the explanation of mental health.

*Research questions 2:* Is each latent and manifest function associated with mental health?

*Research questions 3:* Which specific components of the latent deprivation model (i.e., which latent and manifest functions) do independently contribute to the explanation of mental health, when the influence of the other components is statistically held constant?

## Methods

### Literature search

The literature search (for further details see Supplementary Material A) was done via the databases *PsycINFO, Scopus, PSYNDEX* and *PubMed*.^1^ Another major source of studies was the archive on unemployment-related psychological literature that is kept at the third author’s department, which is regularly updated and includes, among other papers, the primary studies used in two former meta-analyses on the psychological consequences of unemployment (Paul and Moser, 2006, 2009). Each potentially useful study was scrutinized for its fit to our inclusion criteria.

### Inclusion criteria

Criteria for inclusion into the meta-analysis were the following: (1) Study published in English or German^2^; (2) Quantitative study reporting data that enable the computation of effect sizes; (3) The study belonged to one of the two following design types: (A) Groups with differing employment status (e.g., employed and unemployed) were compared with regard to at least one latent function; (B) At least one latent function was correlated with a measure for mental health.

### Operationalizations

#### Employment status

In order to ascertain a sufficient homogeneity of the unemployed groups in the meta-analysis, a sample was only coded as “unemployed” if it matched one of the following criteria: (1) Participants were officially registered as unemployed; (2) participants were explicitly described as being involuntarily out of work; (3) participants were described as having lost their jobs during the last 3 years in a way that implies involuntariness (e.g., through a factory closure); (4) unemployed participants were sufficiently distinguished from students, homemakers and retirees, e.g., by reporting results separately for the four groups.

For the “employed” category, all groups described as being “employed” or “in paid work” or with a similar formulation were accepted. We included not only permanent full-time employment, but also other forms of employment, such as part-time or temporary employment and self-employment. Groups of formerly unemployed people who had found new jobs were also included.

The “out of the labor force” (OLF)-category encompasses all persons who do not participate in the labor market, meaning that they are out of employment but also have currently no intention to take up a job (Rengers, 2004). In the current meta-analysis, these were students, homemakers, and retirees. Whenever a group of participants was described with one of these terms in a primary study, it was coded as OLF.^3^

#### Latent and manifest functions

We included all measures of the latent and manifest functions that sufficiently matched Jahoda’s respective concepts. In most cases, the scales used in the primary studies were explicitly developed to measure the dimensions of her model. One of the most frequently used measures were the Access to Categories of Experience (ACE)-scales (Miles, 1983; Evans, 1986), which measure the latent functions with three items per dimension, in combination with Ullah’s (1990) four-items scale for financial strain, measuring the manifest function of employment. Another frequently used measure were the Latent and Manifest Benefits (LaMB)-scales which measure each of the latent and manifest functions with six items (Muller et al., 2005). Note, that for the manifest function, data were reverse coded such that higher values denote a higher level of fulfillment of the manifest function.

We also included some studies that did not explicitly mention Jahoda or the latent deprivation theory, but used scales with a content equivalent to Jahoda’s functions of employment. For example, several studies used measures for social support including a subscale asking for the frequency of social contact, which fits to Jahoda’s concept of social contact. Finally, some primary studies did not analyze the latent functions individually, but used an overall measure of latent deprivation instead (e.g., Brief et al., 1995). These studies were also included in the present meta-analysis and used in a separate analysis.

#### Mental health

We used five indicator variables of mental health: Mixed symptoms of distress, depression, anxiety, self-esteem, and subjective well-being/life satisfaction. These variables have successfully been used in former meta-analyses on the mental health effects of unemployment (e.g., Paul and Moser, 2009). A principal component analysis based on a meta-analytically derived intercorrelation matrix resulted in a clear one-factor solution, with all five of these variables having high loadings on this factor (Paul and Moser, 2009). We therefore believe it is appropriate to combine individual effect sizes for these variables into an overall-effect size for general mental health.

The most frequently used measures for the five indicator variables were the *General Health Questionnaire* (Goldberg and Hillier, 1979) for mixed symptoms of distress, the *Beck Depression Inventory* (Beck et al., 1961) and the *Depressive Affect Scale* (Rosenberg, 1965) for depression. For the measurement of anxiety symptoms, the *Affective Well-Being Scale* (Warr and Jackson, 1987) and the *Anxiety Scale* (Zung, 1965) were most often used. Subjective well-being was most frequently measured with the *Life Satisfaction Scale* (Diener et al., 1985) and self-esteem was usually measured with the respective scale developed by Rosenberg (1965).^4^

### Coding procedures

Usually, the coding of study characteristics and the computation of effect sizes was done on the level of the complete study. In a few instances, when the relevant data was reported on the level of subgroups that were relevant for moderator tests [e.g., separately for women and men in Bryce and Haworth (2003)], all information was coded on the level of these subgroups in order to strengthen the power of the moderator tests. In case of longitudinal studies, we used only one measurement point in order to avoid statistically dependent effect sizes entering the meta-analytic computations. In most cases, the first measurement point was used, because at this time the sample size is usually highest in longitudinal studies. The first measurement point also has the advantage of avoiding retest-artifacts that result from repeated measurement of the same people (Ormel et al., 1989).

All data used in the meta-analysis were coded independently by two research assistants, who received a coding training and a short manual, describing typical coding problems and recommended solutions. Interrater-agreement was measured with Kappa for categorical variables and with intraclass coefficients for metric variables. Generally, interrater agreement was high, with an average intraclass coefficient of ICC = 0.97 in the dataset for group comparisons and ICC = 0.96 in the dataset for correlations and an average Kappa of κ = 0.92 in both datasets. The few instances of disagreement between coders were either directly corrected when the reason for the disagreement was an obvious error, or were discussed with the first author until a satisfactory consensus solution was reached.

### Statistical methods

We used the standardized mean difference *d* as measure of effect size for the group comparisons. Whenever possible, *d* was computed from means and standard deviations for the respective groups that were compared with each other. When means and standard deviations were not reported, *d* was estimated from *t*-values, *F*-values, Odds Ratios, frequency tables, *p*-values or correlations, following the recommendations in Lipsey and Wilson (2001).

The association between fulfillment of the latent and manifest functions of employment and mental health was usually reported as the Pearson correlation *r* in the primary studies. When other statistics were reported, we transformed them into *r* with the methods recommended in Lipsey and Wilson (2001). In a few cases, when only the results for a multiple regression were reported, we also included β-coefficients in the meta-analysis, using the correction formula provided by Peterson and Brown (2005), p. 180). For further meta-analytic computations, the correlations were Fisher-Z-transformed, as recommended by Lipsey and Wilson (2001). When results for more than one measure of mental health were reported in the primary study (e.g., for depression and anxiety), we used the average of the two respective correlations (Hunter and Schmidt, 1990).

Finally, the computation of an average meta-analytic effect size was done with the SPSS-macros provided in Lipsey and Wilson (2001, pp. 208–220), using the method of moments as estimation method. Moderator tests were also conducted with these macros.

## Results

### Sample of studies

Altogether, 106 primary studies formed the database of the present meta-analysis (For a list of the primary studies, see Supplementary material F). Of these, 46 studies with 56 independent samples contributed to the analysis for group comparisons with *N* = 48.414 research participants. Furthermore, 88 studies with 134 independent samples were included in the analysis regarding the association between latent and manifest functions of employment and mental health, with *N* = 47.232 participants. Among the studies in the group-comparison data-set, 85.7% were published in peer-reviewed journals, while the remaining studies were published as book chapters, dissertation or diploma theses, or gray literature (e.g., unpublished research reports). Among the studies reporting correlations with mental health, the proportion of peer-reviewed papers was 91.8%. The studies were published between 1980 and 2019. The country contributing the highest number of studies to the datasets was Australia (23% of group-comparison studies and 30% of correlation studies), followed by the UK (14% and 15%), the USA (11% and 13%), Germany (9% and 8%), and Sweden (13% and 4%). Smaller numbers of studies originated in Austria, Belgium, Canada, Croatia, Italy, Israel, Japan, Finland, Lithuania, Netherlands, Norway, Portugal, and Turkey. One study collected data in six different northern countries. In summary, the data included in this meta-analysis came mainly from Australia, North America, and (western) Europe, with only few studies coming from other parts of the world.

### Differences between employment status groups

We found significant differences between employed and unemployed people for all latent functions as well as for the manifest function (see Table 1), with employed persons always reporting a higher level of fulfillment of the latent and manifest functions than unemployed persons. With *d* = 0.94 (95% CI: 0.47; 1.42), the largest effect size emerged for status, followed by *d* = 0.59 (95% CI: 0.35; 0.83) for time structure, *d* = 0.51 (95% CI: 0.29; 0.72) for activity, *d* = 0.45 (95% CI: 0.26; 0.65) for collective purpose and *d* = 0.39 (95% CI: 0.26; 0.52) for social contact. Thus, one of the effect sizes was large, while the others were of medium or small-to-medium size.

**TABLE 1:** Latent and manifest functions of employment–differences between employed and unemployed persons.

Function of employment	*k*	*n*	*d*	*SEd*	*95% CI*	*P*	*Q*	*H*
Combined latent functions	47	32,165	0.61	0.0834	0.44; 0.77	0.0000	1135.79***	4.97
OALF	5	1,356	1.26	0.0654	1.13; 1.39	0.0000	2.28	0.76
Time structure	22	7,335	0.59	0.1231	0.35; 0.83	0.0000	359.15***	4.14
Collective purpose	23	13,648	0.45	0.0991	0.26; 0.65	0.0000	400.78***	4.27
Social contact	34	16,836	0.39	0.0673	0.26; 0.52	0.0000	339.15***	3.21
Status	19	7,245	0.94	0.2432	0.47; 1.42	0.0001	1147.88***	7.99
Activity	30	28,108	0.51	0.1097	0.29; 0.72	0.0000	976.08***	5.80
Manifest function	19	18,044	0.73	0.1382	0.46; 1.01	0.0000	922.02***	7.16

Combined latent functions, average of the effect sizes for each sample; OALF (Overall latent functions), study used a single scale measuring all latent functions at once; Manifest function, financial strain (reverse coded); *k*, number of effect sizes; *n*, combined sample size; *d*, average effect size (Random Effects); *SEd*, standard error of *d*; *95% CI*, 95% confidence interval of *d*; *P*, significance level of *d*; *Q*, heterogeneity statistic; *H*, descriptive heterogeneity statistic; ***p < 0.001.

When we combined the effects for the five latent functions into one overall effect for each study (by computing the average of the individual effect sizes per study), a medium size effect was found *d* = 0.61 (95% CI: 0.44; 0.77). Furthermore, a few primary studies did not scrutinize the individual latent functions separately, but used an overall-measure of latent deprivation instead. For these studies, a large average effect size emerged *d* = 1.26 (95% CI: 1.13; 1.39).

Finally, the employed individuals also reported a better financial situation than the unemployed individuals. This effect was of medium-to-large size: *d* = 0.73 (95% CI: 0.46; 1.01). Thus, employed persons did not only have more access to the latent functions of employment, they also reported a higher level of fulfillment of the manifest function.

Only few studies reported comparisons between employed people and people who were out of the labor force (see Table 2). Nevertheless, with at least *k* = 6 samples and *n* = 1732 participants (for status), the data base was large enough for every function of employment to justify a meta-analytic integration.

**TABLE 2:** Latent and manifest functions of employment–differences between employed persons and persons who are out of the labor force (OLF).

Function of employment	*k*	*n*	*d*	*SEd*	*95% CI*	*P*	*Q*	*H*
Combined latent functions	13	3,340	0.26	0.1015	0.06; 0.46	0.0104	92.77***	2.78
Time structure	8	1,958	0.36	0.1778	0.01; 0.71	0.0442	91.44***	3.61
Collective purpose	7	1,962	0.35	0.1464	0.06; 0.63	0.0182	54.26***	3.01
Social contact	9	2,600	0.26	0.1693	–0.07; 0.59	0.1222	127.89***	4.00
Status	6	1,732	0.11	0.0478	0.02; 0.20	0.0207	3.94	0.89
Activity	7	2,263	0.18	0.2643	–0.34; 0.70	0.4904	201.29***	5.79
Manifest function	7	6,503	0.15	0.1048	–0.05; 0.36	0.1406	79.11***	3.63

Combined latent functions, average of the effect sizes for each sample; *k*, number of effect sizes; *n*, combined sample size; *d*, average effect size (Random Effects); *SEd*, standard error of *d*; *95% CI*, 95% confidence interval of *d*; *P*, significance level of *d*; *Q*, heterogeneity statistic; *H*, descriptive heterogeneity statistic; ***p < 0.001.

The general trend showed employed people reporting more latent functions than OLF-people did, as was expected. However, effect sizes were generally small and not significant for some of the latent functions. Significant group differences were identified for time structure, with *d* = 0.36 (95% CI: 0.01; 0.71), for collective purpose with *d* = 0.35 (95% CI: 0.06; 0.63), and for status, with *d* = 0.11 (95% CI: 0.02; 0.20). The weak effects for social contact (*d* = 0.26; 95% CI: −0.07; 0.59), and for activity (*d* = 0.18; 95% CI: −0.34; 0.70) were not significant. Reflecting the pattern of generally small differences between employed and OLF persons, the effect size for the overall effect, combining the five individual latent functions, was also small: *d* = 0.26 (95% CI: 0.06; 0.46).

Finally, we found no difference for the manifest function (*d* = 0.15; 95% CI: −0.05; 0.36), meaning that employed persons and OLF persons did not differ with regard to their financial situation.

In the next step, we meta-analyzed the comparisons between employed people and the three specific subgroups of the OLF-category, i.e., students, homemakers, and retirees. For reasons of limited space and because these analyses were often based on small sample sizes, we will give only a short summary here (see Supplementary Tables 3–5):

The overall difference between employed people and students was significant but small, with employed people reporting slightly more latent functions than students (*d* = 0.18, 95% CI: 0.01; 0.35). Specifically, students had less time structure (*d* = 0.38, 95% CI: 0.25; 0.52) and less collective purpose (*d* = 0.36, 95% CI: 0.11; 0.62) than employed people. They also reported a worse financial situation (*d* = 0.30, 95% CI: 0.08; 0.53).

The overall difference between employed people and homemakers was small and not significant (*d* = 0.25, 95% CI: −0.12; 0.62). There also were no significant differences with regard to any of the specific dimensions.

In contrast, retired people experienced considerably less latent functions than employed people, with an effect size about as large as the difference between employed and unemployed people (*d* = 0.70, 95% CI: 0.44; 0.96). The retirees reported significantly less social contact (*d* = 1.05, 95% CI: 0.95; 1.15), less activity (*d* = 0.97, 95% CI: 0.63; 1.32), less collective purpose (*d* = 0.56, 95% CI: 0.21; 0.91) as well as less status (*d* = 0.16, 95% CI: 0.07; 0.25) than the employees.

Furthermore, for the comparison of unemployed people and OLF people, an overall small effect size emerged (*d* = −0.35; 95% CI: −0.53 to −0.17), with unemployed persons reporting significantly less latent functions than OLF persons did (see Table 3). With regard to the specific latent functions, unemployed people were significantly deprived for status, time structure, and collective purpose in comparison to OLF people. The effect for status was of small-to-medium size (*d* = −0.56; 95% CI: −0.87 to −0.24), while the effects for time structure (*d* = −0.39; 95% CI: −0.69 to −0.10), and collective purpose were small (*d* = −0.32; 95% CI: −0.58 to −0.05). No significant differences were found for activity and social contact. Unemployed people were also characterized by a worse financial situation compared to OLF people, as reflected in the medium effect size of *d* = −0.47 (95% CI: −0.87 to −0.06).

**TABLE 3:** Latent and manifest functions of employment–differences between unemployed persons and persons who are out of the labor force (OLF).

Function of employment	*k*	*n*	*d*	*SEd*	*95% CI*	*P*	*Q*	*H*
Combined latent functions	12	1,452	–0.35	0.0917	–0.53; –0.17	0.0001	35.05***	1.78
Time structure	8	877	–0.39	0.1489	–0.69; –0.10	0.0081	37.14***	2.30
Collect. purpose	7	845	–0.32	0.1354	–0.58; –0.05	0.0185	27.71***	2.03
Social contact	9	1,139	–0.21	0.1354	–0.47; 0.06	0.1233	42.45***	2.46
Status	6	460	–0.56	0.1627	–0.87; -0.24	0.0006	18.85**	1.94
Activity	7	790	–0.26	0.2332	–0.72; 0.19	0.2588	69.20***	3.40
Manifest functions	7	4,350	–0.47	0.2079	–0.87; –0.06	0.0249	161.96***	5.69

Combined latent functions, average of the effect sizes for each sample; *k*, number of effect sizes; *n*, combined sample size; *d*, average effect size (Random Effects); *SEd*, standard error of *d*; *95% CI*, 95% confidence interval of *d*; *P*, significance level of *d*; *Q*, heterogeneity statistic; *H*, descriptive heterogeneity statistic; **p < 0.01, ***p < 0.001.

Further comparisons showed that unemployed people reported less latent functions than the specific OLF-subgroups, although the sizes of the effects varied (see Supplementary Tables 6–8). For students, the effect was of small-to medium size for the overall comparison (*d* = −0.39; 95% CI: −0.66 to −0.11). They reported significantly more time structure, (*d* = −0.54; 95% CI: −0.88 to −0.19), social contact (*d* = −0.51; 95% CI: −1.01 to −0.01), and status (*d* = −0.47; 95% CI: −0.73 to −0.21) than unemployed people.

For homemakers, the overall effect was of medium size (*d* = −0.48; 95% CI: −0.64 to −0.32). With the exception of social contact, homemakers had better access to all latent functions than unemployed people (activity: *d* = −0.76; 95% CI: −1.28 to −0.24; status: *d* = −0.71; 95% CI: −1.00 to −0.43; collective purpose: *d* = −0.45; 95% CI: −0.63 to −0.27; time structure: *d* = −0.37; 95% CI: −0.63 to −0.12).

For retirees, the overall difference was of small size, favoring the retirees: (*d* = −0.17; 95% CI: −0.31 to −0.03). They reported significantly more status (*d* = −0.72; 95% CI: −1.04 to −0.41), and collective purpose (*d* = −0.25; 95% CI: −0.39 to −0.11) than unemployed people. Social contact was the only latent function for which unemployed people showed a significantly better fulfillment compared to retirees (*d* = 0.24; 95% CI: 0.00–0.48).

Finally, unemployed people also reported to be in a worse financial situation than students (*d* = −0.41; 95% CI: −0.53 to −0.30), homemakers (*d* = −0.46; 95% CI: −0.92 to −0.01), and retirees (*d* = −0.83; 95% CI: −1.58 to −0.07).

### Associations of latent and manifest functions of employment and mental health

The average correlation between the combined latent functions and mental health was positive and of medium size (see Table 4). For the association between the overall value of the latent functions and overall mental health, the correlation was *r* = 0.28 (95% CI 0.25–0.32). For those primary studies using an undifferentiated measure of the latent functions a slightly larger association was detected: *r* = 0.43 (95% CI 0.34–0.53).

**TABLE 4:** Correlations between functions of employment and mental health.

Function of employment	*k*	*n*	*r*	*SEr*	*95% CI*	*P*	*Q*	*H*
Combined latent functions	100	30,044	0.28	0.0179	0.25; 0.32	0.0000	790.44***	2.83
OALF	16	7,600	0.43	0.0467	0.34; 0.52	0.0000	210.40***	3.75
Time structure	55	16,093	0.25	0.0263	0.20; 0.30	0.0000	519.31***	3.10
Collective purpose	41	14,203	0.31	0.0289	0.25; 0.37	0.0000	409.65***	3.20
Social contact	60	18,563	0.23	0.0214	0.19; 0.28	0.0000	400.04***	2.60
Status	43	15,879	0.28	0.0294	0.23; 0.34	0.0000	489.63***	3.41
Activity	49	15,896	0.21	0.0217	0.17; 0.25	0.0000	281.60***	2.42
Manifest function	68	27,092	0.28	0.0328	0.22; 0.35	0.0000	1753.90***	5.12

OALF (Overall latent functions), study used a single scale measuring all latent functions at once; *n*, combined sample size; *r*, average meta-analytic correlation (Random Effects); *SEr*, standard error of *r*; *95% CI*, 95% confidence interval of *r*; *P*, significance level of *r*; *Q*, heterogeneity statistic; *H*, descriptive heterogeneity statistic; ***p < 0.001.

Looking at individual latent functions, the largest correlation was found for collective purpose (*r* = 0.31, 95% CI 0.25–0.37), followed by status (*r* = 0.28, 95% CI 0.23–0.34), time structure (*r* = 0.25, 95% CI 0.20–0.30), social contact (*r* = 0.23, 95% CI 0.19–0.28), and activity (*r* = 0.21, 95% CI 0.17–0.25). Thus, the average effect sizes for all latent functions were significant and of medium or small-to-medium size.

With *r* = 0.28 (95% CI 0.19–0.28), the meta-analytic correlation between a person’s financial situation and his or her mental health was also of medium size and identical to the average correlation that was found for the combined measure of the five latent functions. In summary, in bivariate analyses all latent functions as well as the manifest function emerged as predictors of mental health. The effect sizes were of small-to-medium or of medium size.

We also checked, whether demographic variables moderated the associations between overall latent functions and mental health as well as between the manifest function and mental health. However, no significant moderator effect was detected for age, gender, relationship status, or years of education (not reported in detail here for reasons of space).

Finally, we tested the influence of the latent and manifest functions on mental health in a multivariate analysis, i.e., when all six dimensions were simultaneously included as predictors in a multiple regression analysis. In order to do this, we first meta-analyzed the intercorrelations of the latent and manifest functions, and used the resulting intercorrelation matrix as input for the regression analysis. Results showed that, together, the latent and manifest functions explained 19% of the variance in mental health (see Table 5). All six manifest and latent functions emerged as significant predictors of mental health in this analysis. The effects were of small-to-medium size, with financial situation β = 0.20, *p* < 0.001, collective purpose β = 0.16, *p* < 0.001, time structure β = 0.15, *p* < 0.001, status β = 0.13, *p* < 0.001, social contact β = 0.05, *p* < 0.001, and activity β = 0.03, *p* = 0.014. Thus, each latent and manifest function showed an independent influence on mental health, when the influence of the other variables in the latent deprivation model was parsed out.

**TABLE 5:** Multiple regression analysis for the prediction of mental health.

Variable	B	SE_B_	ß	*t*	*P*
Constant	0.000	0.009	–	0.00	1.000
Time structure	0.149	0.010	0.149	15.30	<0.001
Collective purpose	0.160	0.011	0.160	15.01	<0.001
Social contact	0.054	0.011	0.054	5.16	<0.001
Status	0.126	0.011	0.126	11.70	<0.001
Activity	0.026	0.011	0.026	2.47	0.014
Manifest function	0.195	0.010	0.195	20.44	<0.001

Adjusted *R*^2^ = 0.194; *N* = 9,495; the smallest specific *N* of the meta-analytically derived intercorrelation matrix was used for multivariate estimation.

## Discussion

The group comparisons that were conducted here generally supported the latent deprivation model’s proposition that employment is the most important provider of the latent functions. However, while employed people clearly reported better access to all five functions than unemployed people did, as expected, the differences between employed people and OLF people were smaller and only significant for time structure, collective purpose, and status.

A closer look at the three subgroups in the OLF-category showed considerable differences between homemakers, students, and retirees. Homemakers did not significantly differ from employed people. For students, access to the latent functions was slightly worse than for employed people, while retirees showed a considerable difference to employed people and a level of latent deprivation that was similar to unemployed people.

Thus, at first glance, one might conclude that alternative life situations exist in modern societies (specifically homemaking and education) that are able to substitute employment as provider of the latent functions to a considerable degree. However, it might be argued that the life situations of students and homemakers are directly and indirectly related to employment, partly explaining the small differences to the employed group: Formal education is an institution that is designed to prepare young people for the world of work and has important similarities with it, such as externally generated goals stimulating the individual to become active, a prescribed time structure, automatic social contact with others, and a (limited) level of status and social recognition. Thus, formal education does not only prepare young people for the world of work, but also mimics it to a certain degree, explaining the relatively large level of latent functions it seems to provide.

Furthermore, homemaking is a life situation that has changed considerably in recent decades. A large majority of countries with developed economies now have regulations for parental leave, providing parents with paid time away from work while the employer is required to guarantee the return to the old job or a similar job after the parental leave has ended (Addati, 2015). Thus, modern homemaking does probably not feel like a permanent separation from one’s job, but more like a limited break. As a consequence, modern homemakers might still experience some latent functions resulting from their job, for example status and collective purpose. This is because they are still perceived (and perceive themselves) as people who have an occupational position that implies doing important things for society and deserving recognition for this, even if they are temporarily away from it.

Finally, “employed people” is in itself a heterogeneous group, and the extent to which the manifest and latent functions are fulfilled might partially depend on the quality of the employment situation. For example, it has recently been reported that the number of weekly working hours is related to both types of functions (Bähr et al., 2022).

In summary, higher education and modern homemaking might have shown small differences to employment, not because they are independent alternative sources of the five latent functions, but because of their close connection to employment. In this case, the high saturation with the latent functions would be an indirect effect of employment. In contrast, retired people, who – on average – probably retain comparatively weak links to the world of paid work, reported levels of the latent functions similar to unemployed people. Thus, the results for OLF-people might be more supportive for Jahoda’s assumptions of employment as the main provider of the latent functions than they appear at first glance.

Regarding the prediction of mental health, the assumptions of the latent deprivation model were largely supported. All six dimensions in the model were incrementally associated with mental health and together they were able to explain about one-fifth of the variance in mental health, which is a noteworthy amount in our eyes. Thus, our conclusion is that despite some correlations between the six dimensions and despite the results in some primary studies, we can clearly recommend that it is of value to assess all dimensions.

Of note is that contrary to a controversial assumption from Jahoda (1982), the negative effect of financial deprivation is not negligible in contemporary societies. On the contrary, its effect was similar to the most influential latent functions. A similar result has recently been reported in Bähr et al. (2022). Thus, Jahoda’s optimism regarding the ability of the modern welfare state to protect from the soul-destroying consequences of poverty (which she herself observed in her famous Marienthal-study, see Jahoda et al., 1933/1975) must be qualified: Many unemployed people appear to still experience a degree of financial difficulties that clearly impedes on their mental health. Possibly, Jahoda was so impressed by the devastating effect of absolute poverty (i.e., the inability to afford basic necessities such as food or clothing), which she witnessed in Marienthal, that she underestimated the negative impact of the relative poverty (i.e., a household income below a certain threshold, e.g., 50% of the median income), which is still prevalent in modern welfare states.

All latent and manifest functions together were able to explain about one fifth of the variance in mental health in this meta-analysis. While this is a considerable amount in our eyes, it still leads to the question what other factors might improve this prediction even further. From a methodological perspective, both intraindividual variability and variability between the employment status groups might be taken into account in future research. For example, processes of unemployment coping and normalization (Houssemand et al., 2020), as well as recent analyses on the heterogeneity of, for ex., employed people with respect to the degree of fulfillment of the latent functions they experience in their work (Bähr et al., 2022) represent these two approaches. Finally, since Jahoda conceptualized her latent functions in analogy to basic human needs, i.e., as psychological inputs that are necessary to stabilize well-being, a look at contemporary need theories might be helpful. Do these theories include need dimensions that are not yet included in the latent deprivation model, but might function in a similar way as the latent functions of employment? A recent study scrutinizing competence, a need dimension derived from self-determination theory (Deci and Ryan, 2000), demonstrated that this variable indeed increased when unemployed people found new jobs and that these changes influenced their mental health. The experience of competence might thus be included in the model as a sixth latent function of employment (Zechmann and Paul, 2019).

In order to check whether the validity of our findings is threatened by the use of non-established scales, or the influence of publications bias, we conducted sensitivity analyses, involving a series of moderator tests and tests for the symmetry of the distribution of effect sizes. We found some evidence that studies using non-standard scales for the measurement of the latent and manifest functions reported somewhat larger effect sizes than studies using established scales. Furthermore, studies in which the latent deprivation model was the main topic of the publication had larger effect sizes than studies in which it was only one topic among others. And finally, we identified some signs of asymmetry for the distributions of effect sizes, pointing toward the influence of publication bias.

Nevertheless, the influence of these distortions was weak, with average effects in all subgroups of the moderator tests remaining clearly significant. The average effects also remaining significant when the studies putatively missing due to publication bias were included through imputation procedures (for details, see Supplementary Material E). In summary, while some signs of bias could be identified, the overall threat to the validity of the results reported here remained limited.

### Limitations

The present meta-analysis incorporates primary studies from 20 different countries, ensuring a certain degree of cross-cultural generalizability of the results. Nevertheless, several important world regions were not represented at all, leading to the question of whether the latent deprivation model is also valid in non-western contexts. Further studies from these parts of the world are needed. With respect to time structure, some adaptation processes (Bähr et al., 2022) or, more generally, reappraisal and “normalization” processes (Houssemand et al., 2020) might play a role. Unfortunately, we were not able to take this further into account by, for example, controlling for how long the respective people held their employment status.

In order to enlarge our database, we included studies that did not explicitly mention Marie Jahoda or her latent deprivation model, but still scrutinized variables that were clearly equivalent to one or more of the dimensions in the model. The equivalence of each of these variables was thoroughly checked, safeguarding the construct validity of the latent and manifest functions. We therefore believe that the inclusion of studies from outside of the established research field did not introduce a bias in our meta-analysis.

Another possible limitation comes from within the research field. The two most frequently used measurement instruments of the latent functions of employment, the access to categories of employment scales (ACE, Miles, 1983), and the latent and manifest benefits scales (LaMB, Muller et al., 2005), yielded slightly differing results in the present meta-analysis. Moderator tests showed that the resulting correlations of social contact, collective purpose, status, and activity with mental health were significantly larger when the ACE-subscale was used, compared to the respective LaMB-subscale. For one dimension – status – this difference was remarkably large, with *r* = 0.44 (95% CI: 0.41–0.47) for the ACE-subscale and *r* = 0.17 (95%CI: 0.14–0.20) for the LaMB-subscale. In general, the reasons for these higher correlations with ACE are presently unclear. They might hint at problems with the construct validity of one of the two most established measurement instruments for the latent functions of employment.

Finally, reporting biases or the use of untested *ad-hoc* scales could have distorted the results. However, see Supplementary material E for sensitivity analyses showing that this is unlikely.

### Implications

Traditionally, the latent deprivation model has been associated with the field of psychological unemployment research. As the present results show, it is indeed helpful in explaining the distress unemployed people typically suffer from (Paul and Moser, 2009). Accordingly, interventions for unemployed people, those aiming at health as well as those aiming at re-employment, further education, or other topics, should be checked for the amount of the latent and manifest functions they provide. For example, an intervention that aims to improve mental health in unemployed people should try to increase the extent to which these functions are fulfilled, because unemployed people are usually deprived of them when compared to other people. Thus, an intervention for unemployed people should have a clear temporal structure, should provide opportunities for contact with other people, and should activate participants (in contrast, for example, to training schemes relying mainly on lecture-style instruction). Such interventions should also try to convey a feeling that the activity is useful to other people and might be valued by society. For example, unemployed people repairing toys for children are likely to experience collective purpose as well as some status (European Social Fund – ESF News, 2022), demonstrating that it might even be possible that interventions for unemployed people do provide all types of latent functions. As another example, interventions that aim at preparing still employed people for retirement (“retirement preparation courses”) should be designed based on the five latent functions, by including specific plans (time structure), or preparing and encouraging activities that are realized in companion with others (social contact) and that are meaningful for others (collective purpose).

However, the latent deprivation model is more than just a theory of unemployment distress. It formulates testable assumptions about the psychological effects of one of the most important institutions of contemporary societies, i.e., employment. As such, it has implications for other important topics that are currently debated in many countries, for example the introduction of a universal basic income. If one assumes – in line with traditional economic thinking – that workers are mainly motivated to show up for work by financial rewards, such an unconditional basic income would probably result in a strong reduction of the labor supply. In contrast, the latent deprivation model assumes work motivation to be, to a large part, determined by non-financial aspects of work, predicting only a weak reduction of the labor supply or no reduction at all. The few existing studies on the consequences of an unconditional basic income support the predictions of the latent deprivation model (de Paz-Báñez et al., 2020). Possible fears concerning a strong reduction of the labor supply (and tax revenues) following the introduction of a universal basic income might therefore be unfounded.

## Data availability statement

The raw data supporting the conclusions of this article will be made available by the authors upon reasonable request.

## Author contributions

KP contributed to the conception and design of the study, analysis of the data, interpretation of the results, and drafting and revisions of the manuscript. HS contributed to the data acquisition, data analysis, interpretation of the data, and helped drafting the manuscript. KM contributed to the conception and design of the study, interpretation of the data, and revision of the manuscript. AZ contributed to the interpretation of the results and revision of the manuscript. BB contributed to the conception of the study and revision of the manuscript. All authors contributed to the article and approved the submitted version.

## Footnotes

1 Search terms combined “unemploy*” with either “Jahoda” or with “(latent* OR manifest*)” or with “(function* OR benefi* OR depriv*)” or with “[(time* OR activ* OR status* OR purpos* or social*) AND (health* OR depress* OR anxi* OR somatiza* OR esteem* OR satisfac* OR mood* OR well being*)]”.

2 We intended to test whether effect sizes differed between studies in English and studies published in another language, in this case German. Such a language bias could have threatened the validity of our results. However, due to the very low number of studies published in German (1, 3%), these tests were not feasible in a meaningful manner.

3 Typically, this meant that students were people in their twenties attending institutions of higher education and retirees were persons beyond the usual line of retirement, i.e., 65 years or older. However, in some cases participants were very young and the “student” group thus consisted of pupils still going to school (e.g., Winefield and Tiggemann, 1985). Furthermore, the group of retirees sometimes included people in early retirement with an age below the usual retirement line (e.g., Read et al., 2017).

4 More detailed information on the measurement instruments used in the primary studies can be found in the Supplementary Tables 1, 2.
