# Supplementary material for: Employment status, psychological needs, and mental health: Meta-analytic findings concerning the latent deprivation model
Source: Front Psychol. 2023 Mar 2;14:1017358. doi: 10.3389/fpsyg.2023.1017358 (PMC10017486; doi:10.3389/fpsyg.2023.1017358)
Supplement: Supplementary file 1 [file Data_Sheet_1.pdf]

Paul, K. I., Scholl, H., Moser, K., Zechmann, A. & Batinic, B. (2023). Employment status, psychological needs, and mental health: Meta-analytic findings concerning the latent deprivation model. *Front. Psychol. Sec. Personality and Social Psychology*, Volume 14 - 2023 | doi: 10.3389/fpsyg.2023.1017358

Supplementary material

A) Literature search - Explanation of search strategy

Because of our extensive knowledge of the literature on both, research on unemployment and mental health and on the latent deprivation model, we used our literature archive for the first step of our search process. This archive was built up during the last two decades, involving several literature searches concerning the psychological consequences of unemployment, in particular for mental health. (The archive is accessible to any visitor of our research group with reasonable scientific interest.) This led to the identification and retrieval of n=92 reports or studies.

This search was complemented by a search of databases (PsycINFO, PSYNDEX, PubMed, Scopus). Because we completed several literature searches in previous years that were the basis for publications on the model of latent and manifest functions (see for ex. Paul & Batinic, 2010; Selenko, Batinic & Paul, 2011), we were confident that earlier research would be already part of our department archive. Thus, the database searches started with the year 2015. The only exception was the search in PubMed, which was not time-limited because this database had only rarely been used in our earlier searches. This search process using databases led to the identification of further n=14 eligible studies. Figure 1 shows the search process in more detail.

Figure 1- Flow diagram for literature search

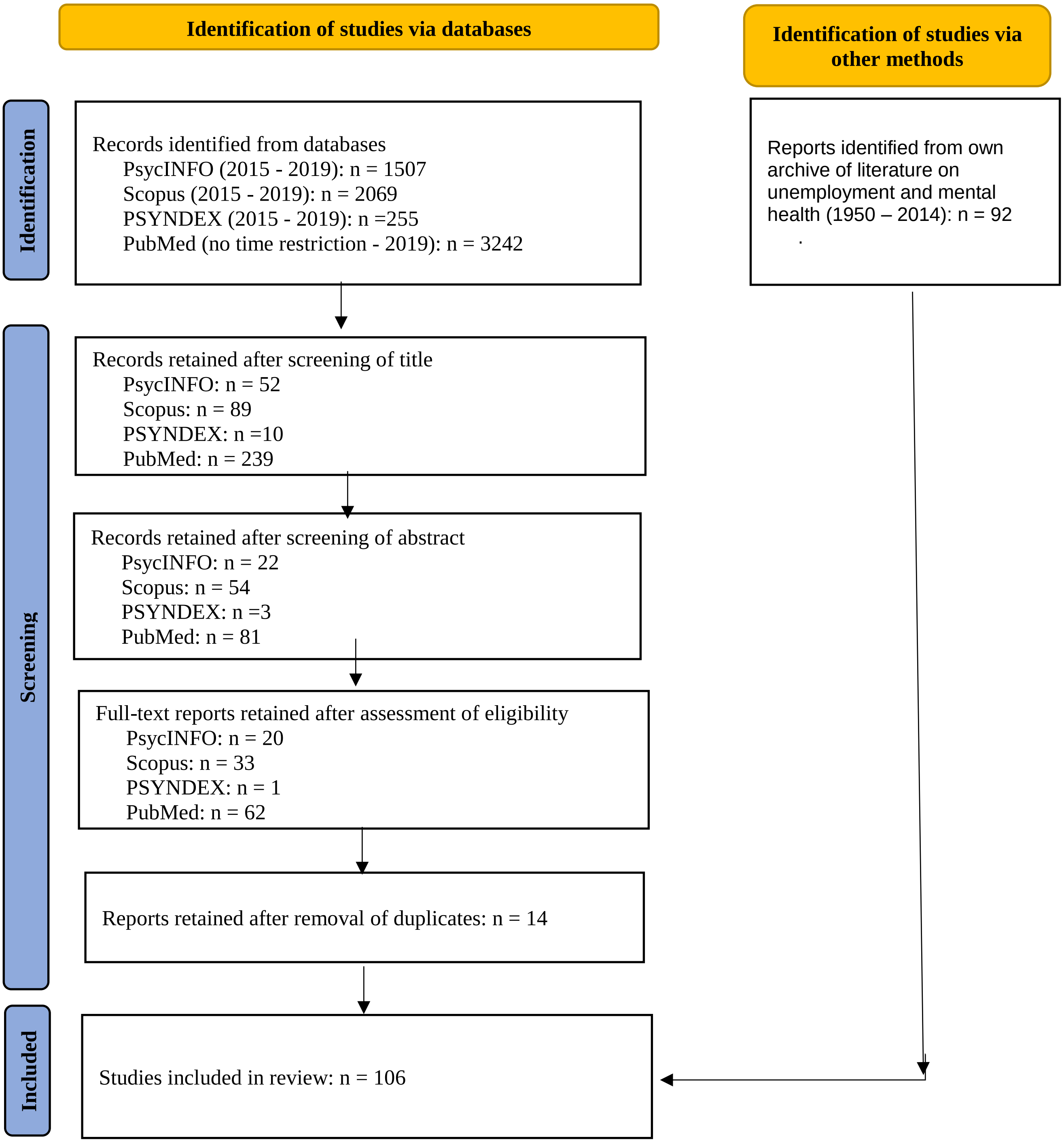

B) Most frequently used measurement instruments

S1 - Scales for the measurement of manifest and latent functions of employment

Function of employment	*Measurement instrument*	*Studies on group comparisons*	*Studies on association with mental health*
Overall latent functions	*ACE Scale* (Evans, 1986)	57.1%	50.0%
	Experiential Deprivation Scale (Brief et al., 1995)	28.6%	25.0%
	LAMB Scale (Muller et al., 2005)	14.3%	5.0%
	Self-constructed scale	-	20.0%
Time structure	Subscale of *ACE Scale* (Evans, 1986)	40.9%	20.6%
	Time Structure Questionnaire (Bond & Feather, 1988)	31.8%	28.6%
	Subscale of *LAMB Scale* (Muller et al., 2005)	22.7%	33.3%
	Subscale of *Use of Time Scale* (Feather & Bond, 1983)	4.5%	6.3%
Status	Subscale of *ACE Scale* (Evans, 1986)	47.6%	25.9%
	Subscale of *LAMB Scale* (Muller et al., 2005)	23.8%	37.0%
	Self-constructed scale	14.3%	18.5%
	Hollingshead Index (1975)	9.5%	3.7%
Social contact	Self-constructed scale	42.5%	32.1%
	Subscale of *ACE Scale* (Evans, 1986)	25.0%	17.9%
	Subscale of *LAMB Scale* (Muller et al., 2005)	12.5%	25.6%
Activity	Self-constructed scale	44.1%	37.5%
	Subscale of *ACE Scale* (Evans, 1986)	29.4%	25.0%
	Subscale of *LAMB Scale* (Muller et al., 2005)	14.7%	31.3%
Collective purpose	Subscale of *ACE Scale* (Evans, 1986)	34.6%	22.6%
	Self-constructed scale	23.1%	17.0%
	Subscale of *LAMB Scale* (Muller et al., 2005)	19.2%	37.7%
	Subscale of *Use of Time Scale* (Feather & Bond, 1983)	7,7%	11.3%
Manifest function	Self-constructed scale	28.0%	27.1%
	Scale fom Ullah (1990)/ Creed & Macintyre (2001)	28.0%	21.2%
	Subscale of *LAMB Scale* (Muller et al., 2005)	12.0%	18.8%
	Financial Stress Scale (Feather, 1989)	12.0%	3.5%

Notes. Scale names denote either complete original scales or derivative/short versions of the original scales. Reported are the percentages of studies in which the scales were used.

S2 - Most frequently used scales for the measurement of mental health

Indicator of mental health	*Scale*	*Frequency*
Mixed symptoms of distress	*GHQ* (Goldberg & Hillier, 1979)	75.9%
	SCL-90 (Derogatis, 1977)	9.6%
	Mental Health Inventory (Berwick et al., 1991)	3.6%
Depression	*BDI* (Beck et al., 1961)	25.0%
	Depressive Affect Scale (Rosenberg, 1965)	13.6%
	CES-D (Radloff, 1977)	11.4%
	Zung Depression Scale (Zung, 1965)	6.8%
Anxiety	*Affective Well-Being Scale* (Warr, 1987)	31.6%
	Zung Anxiety Scale (Zung, 1965)	15.8%
	STAI (Spielberger, 1983)	10.5%
	Self-constructed scale	10.5%
Subjective well-being / Life satisfaction	*Diener Life Satisfaction Scale* (Diener et al., 1985)	43.8%
	Warr Life Satisfaction Scale (Warr et al., 1979)	25.0%
	Single item	12.5%
Self Esteem	*Rosenberg-Scale* (Rosenberg, 1965)	63.9%
	Single item	13.9%
	Global Self Worth Subscale (Messer & Harter, 1986)	8.3%

Note. Scale names denote either complete original scales or derivative/short versions of the original scales.

C) Comparisons between employed people and students, homemakers, and retirees for latent and manifest functions

S3 - Latent and manifest functions of employment - differences between employed persons and students

Function of employment	*k*	*n*	*d*	*SEd*	*95% CI*	*p*	*Q*	*H*
Combined latent functions	9	3204	0.18	0.0872	0.01; 0.35	0.0347	24.64**	1.76
Time structure	5	2091	0.38	0.0679	0.25; 0.52	0.0000	2.77	0.83
Collective purpose	4	2095	0.36	0.1320	0.11; 0.62	0.0058	11.53**	1.96
Social contact	6	2733	-0.10	0.0780	-0.25; 0.05	0.2020	10.08	1.42
Status	4	2116	0.12	0.1070	-0.09; 0.33	0.2556	6.78	1.50
Activity	5	2647	-0.04	0.2235	-0.48; 0.40	0.8619	63.79***	3.99
Manifest function	6	6089	0.30	0.1133	0.08; 0.53	0.0073	40.02***	2.83

Notes. Combined latent functions = average of the effect sizes for each sample; *k* = number of effect sizes; *n* = combined sample size; *d* = average effect size (Random Effects); *SEd* = Standard error of *d*; *95% CI* = 95% confidence interval of *d*; *p* = significance level of *d*; *Q* = heterogeneity statistic; *H* = descriptive heterogeneity statistic; ** $p < 0.01$, *** $p < 0.001$

S4 - Latent and manifest functions of employment - differences between employed persons and homemakers

Function of employment	*k*	*n*	*d*	*SEd*	*95% CI*	*p*	*Q*	*H*
Combined latent functions	4	1988	0.25	0.1885	-0.12; 0.62	0.1917	18.50***	2.48
Time structure	4	1988	0.22	0.2503	-0.27; 0.71	0.3875	33.03***	3.32
Collective purpose	4	1988	0.26	0.1746	-0.08; 0.60	0.1442	15.84**	2.30
Social contact	4	1988	0.64	0.3525	-0.05; 1.33	0.0683	64.16***	4.62
Status	3	1737	0.02	0.0857	-0.14; 0.19	0.7839	0.10	0.22
Activity	3	1737	0.34	0.2182	-0.09; 0.76	0.1243	10.37**	2.28
Manifest function	4	5543	0.15	0.1554	-0.16; 0.45	0.3428	32.77***	3.31

Notes. Combined latent functions = average of the effect sizes for each sample; *k* = number of effect sizes; *n* = combined sample size; *d* = average effect size (Random Effects); *SEd* = Standard error of *d*; *95% CI* = 95% confidence interval of *d*; *p* = significance level of *d*; *Q* = heterogeneity statistic; *H* = descriptive heterogeneity statistic; ** $p < 0.01$, *** $p < 0.001$

S5 - Latent and manifest functions of employment - differences between employed persons and retirees

Function of employment	*k*	*n*	*d*	*SEd*	*95% KI*	*p*	*Q*	*H*
Combined latent functions	4	2289	0.70	0.1321	0.44; 0.96	0.0000	11.95**	2.00
Time structure	4	2289	0.69	0.3516	-0.00; 1.37	0.0512	95.44***	5.64
Collective purpose	4	2289	0.56	0.1789	0.21; 0.91	0.0016	23.24***	2.78
Social contact	4	2289	1.05	0.0496	0.95; 1.15	0.0000	1.28	0.65
Status	4	2289	0.16	0.0471	0.07; 0.25	0.0006	1.36	0.67
Activity	4	2289	0.97	0.1735	0.63; 1.32	0.0000	19.82**	2.57
Manifest function	2	2174	0.04	0.1630	-0.28; 0.36	0.7937	10.44**	3.23

Notes. Combined latent functions = average of the effect sizes for each sample; *k* = number of effect sizes; *n* = combined sample size; *d* = average effect size (Random Effects); *SEd* = Standard error of *d*; *95% CI* = 95% confidence interval of *d*; *p* = significance level of *d*; *Q* = heterogeneity statistic; *H* = descriptive heterogeneity statistic; ** $p < 0.01$, *** $p < 0.001$

D) Comparisons between unemployed people and students, homemakers, and retirees for latent and manifest functions

S6 - Latent and manifest functions of employment - differences between unemployed persons and students

Function of employment	*k*	*n*	*d*	*SEd*	*95% KI*	*p*	*Q*	*H*
Combined latent functions	8	1326	-0.39	0.1400	-0.66; -0.11	0.0057	37.41**	2.31
Time structure	5	778	-0.54	0.1749	-0.88; -0.19	0.0022	18.89***	2.17
Collective purpose	4	746	-0.27	0.1764	-0.62; 0.08	0.1255	13.50**	2.12
Social contact	6	1040	-0.51	0.2542	-1.01; -0.01	0.0442	64.62***	3.60
Status	4	627	-0.47	0.1323	-0.73; -0.21	0.0004	6.31	1.45
Activity	5	957	-0.31	0.3357	-0.97; 0.34	0.3491	82.55***	4.54
Manifest function	5	3932	-0.41	0.0570	-0.53; -0.30	0.0000	7.02	1.33

Notes. Combined latent functions = average of the effect sizes for each sample; *k* = number of effect sizes; *n* = combined sample size; *d* = average effect size (Random Effects); *SEd* = Standard error of *d*; *95% CI* = 95% confidence interval of *d*; *p* = significance level of *d*; *Q* = heterogeneity statistic; *H* = descriptive heterogeneity statistic; ** $p < 0.01$, *** $p < 0.001$

S7 - Latent and manifest functions of employment - differences between unemployed persons and homemakers

Function of employment	*k*	*n*	*d*	*SEd*	*95% KI*	*p*	*Q*	*H*
Combined latent functions	4	710	-0.48	0.0802	-0.64; -0.32	0.0000	0.28	0.31
Time structure	4	710	-0.37	0.1290	-0.63; -0.12	0.0039	6.81	1.51
Collective purpose	4	710	-0.45	0.0924	-0.63; -0.27	0.0000	3.75	1.12
Social contact	4	710	-0.15	0.1996	-0.54; 0.24	0.4532	16.44***	2.34
Status	3	444	-0.71	0.1463	-1.00; -0.43	0.0000	3.32	1.29
Activity	3	444	-0.76	0.2655	-1.28; -0.24	0.0044	10.55**	2.30
Manifest function	4	3461	-0.46	0.2322	-0.92; -0.01	0.0456	58.14***	4.40

Notes. Combined latent functions = average of the effect sizes for each sample; *k* = number of effect sizes; *n* = combined sample size; *d* = average effect size (Random Effects); *SEd* = Standard error of *d*; *95% CI* = 95% confidence interval of *d*; *p* = significance level of *d*; *Q* = heterogeneity statistic; *H* = descriptive heterogeneity statistic; ** $p < 0.01$, *** $p < 0.001$

S8 - Latent and manifest functions of employment - differences between unemployed persons and retirees

Function of employment	*k*	*n*	*d*	*SEd*	*95% KI*	*p*	*Q*	*H*
Combined latent functions	4	975	-0.17	0.0707	-0.31; -0.03	0.0177	0.97	0.57
Time structure	4	975	-0.07	0.2116	-0.48; 0.35	0.7485	16.96***	2.38
Collective purpose	4	975	-0.25	0.0708	-0.39; -0.11	0.0004	1.93	0.80
Social contact	4	975	0.24	0.1220	0.00; 0.48	0.0486	5.79	1.39
Status	4	975	-0.72	0.1629	-1.04; -0.41	0.0000	9.27*	1.76
Activity	4	975	-0.20	0.1398	-0.47; 0.08	0.1605	7.33	1.56
Manifest function	2	889	-0.83	0.3845	-1.58; -0.07	0.0312	21.19***	4.60

Notes. Combined latent functions = average of the effect sizes for each sample; *k* = number of effect sizes; *n* = combined sample size; *d* = average effect size (Random Effects); *SEd* = Standard error of *d*; *95% CI* = 95% confidence interval of *d*; *p* = significance level of *d*; *Q* = heterogeneity statistic; *H* = descriptive heterogeneity statistic; * $p < 0.05$, *** $p < 0.001$

E) Sensitivity analyses

Our sensitivity analyses include three elements: (a) We conducted a moderator test checking whether the size of the effects found in the primary studies was different for studies using established scales vs. newly developed ad-hoc scales; (b) we tested whether the latent deprivation model being the main topic of the respective primary study influenced the size of the effects; (c) we tested for publication bias.

(a) First, we tested the moderating effect of the type of the measure that was used to assess the latent and manifest functions of employment. To do this, we differentiated between established scales such as the ACE-Scales (Evans, 1986) or the LaMB-scales (Muller et al., 2005) that have been frequently used in the research field, and not-established scales that were self-constructed by the authors of the respective study in an ad-hoc manner.
The results (see tables S9 and S10) showed that for the comparisons of employed and unemployed people, effects were significantly stronger ($p < .001$) when status, activity, or the manifest function had been measured with a self-constructed scale compared to an established scale. No effect was found for social contact (for the other functions there was not enough information available to conduct a meaningful test).
Scale type also played a role regarding the size of the correlations with mental health. The correlations were stronger for status and activity when these latent functions had been measured with self-constructed scales in comparison to established scales ($p < .001$). For social contact, however, we found a significant moderator effect in the opposite direction: The average correlation with mental health was stronger, when social contact was measured with an established scale compared to a self-constructed scale ($p < .001$).
Yet, despite these significant moderator effects, the differences between employed and unemployed people as well as the correlations between manifest and latent functions and mental health remained always significant, regardless which type of scale was used.

(b) Second, in order to test whether reporting bias influenced the meta-analytic results, we conducted a moderator analysis for the latent deprivation model being the main topic of the respective primary study or not. In order to operationalize that, we coded whether the latent deprivation model or Marie Jahoda were explicitly named in the title of the publication.
For comparisons of employed and unemployed people, these moderator tests were significant for the combined latent functions, for social contact, status, activity, and for the manifest function ($p < .001$, see tables S11 and S12). In all analyses, the difference between employed and unemployed individuals was larger when the deprivation model or Jahoda herself were directly referenced in the publication title.
For the correlations between latent and manifest functions and mental health, results were mixed: Combined latent functions ($p < .001$), collective purpose ($p < .01$), social contact ($p < .001$) and status

($p < .05$) were found to be more strongly correlated to mental health when Jahoda or her theory were named in the title. For time structure ($p < .01$), activity ($p < .001$), and the manifest function ($p < .05$), the opposite was the case.
However, for group comparisons as well as correlations with mental health, the average effect size always remained significantly different from zero, regardless of whether Jahoda's theory was the main topic of the paper or not.

(c) Finally, we used Egger's regression test and the trim-and-fill method to test for publication bias that might have distorted the results.
For the comparison of unemployed and employed people, Egger's test was not significant with regard to the combined latent functions (intercept = .343, t = 1.327, $p = .191$), indicating no identifiable publication bias. It was also not significant for time structure, collective purpose, social contact, status, activity, or financial situation ($p > .11$ in all analyses). Yet, results for the trim-and-fill-analysis were less consistent: We found no signs of missing studies for the analysis of the combined latent functions and for status. But for the other individual latent functions the trim-and-fill-method suggested the imputation of additional studies in order to adjust for the asymmetry of the distribution of effect sizes. The number of suggested studies for imputation ranged from four (financial situation) to nine (activity), always leading to a reduction in the strength of the average effect. In the most extreme case, i.e. activity, the resulting average effect size would have been reduced to $d = .24$ (95%*CI*: 0.01 - 0.47). However, for all analyses including imputed studies, the difference between employed and unemployed people remained significant.
With regard to the correlations of the combined latent functions to mental health, Egger's test was significant ($p < .001$), indicating asymmetry of the distribution of effect sizes. The trim-and-fill method suggested imputation of 15 hypothetical studies to the original 100 studies, which would have reduced the average correlation to $r = .23$ (95%*CI*: 0.19 - 0.27). For the individual latent functions as well as the manifest function, Egger's test was also significant. The trim-and-fill-method, however, did not indicate asymmetry for time structure, status, and activity. For the other functions, the imputation of six (social contact), twelve (collective purpose), and 15 (financial situation) studies was suggested. Nevertheless, while these imputations would have reduced the average effect sizes to some degree, they would not have led to an insignificant average correlation for any of the functions of employment.

In summary, our sensitivity analyses hinted at a possible slight artificial inflation of the effect sizes due to some authors using self-constructed ad-hoc scales (where the exact methods used to construct these scales usually remain unknown, resulting in questionable psychometric qualities). We also found some evidence that a reporting bias might exist, leading to larger effect sizes being published in papers that explicitly focus on the latent deprivation model. Furthermore, some of the symmetry tests indicated the existence of asymmetry and thus possible publication bias.

In must be added, however, that the resulting distortions of the validity of our findings appear to be limited, because when missing studies were imputed or when the average effect was computed only with studies not explicitly mentioning the latent deprivation model in their titles, the average effect sizes always remained significant and were usually only slightly reduced in their size. Also, the fact that we included a large number of studies that were not mainly concerned with Jahoda’s model in our meta-analysis probably helped to reduce reporting bias.

S9 - Scale type (established vs. self-constructed) as moderator of differences between employed and unemployed persons

Function of employment	*Scale type*	Q_b	*k*	*n*	*d*	*95% CI*	*p*	Q_w
Social contact	est.	3.21	23	10657	0.41	0.36; 0.45	0.0000	298.40
	s-c		10	5677	0.33	0.26; 0.40	0.0000	37.54
Status	est.	189.14***	17	6536	0.41	0.35; 0.47	0.0000	421.01
	s-c		2	709	2.31	2.04; 2.57	0.0000	537.73
Activity	est.	32.87***	18	9825	0.39	0.34; 0.44	0.0000	822.67
	s-c		12	18283	0.60	0.55; 0.65	0.0000	120.54
Manif. funkt.	est.	59.06***	17	14730	0.45	0.41; 0.49	0.0000	862.82
	s-c		2	3314	0.87	0.77; 0.97	0.0000	0.15

Scale type: est. = established scale, s-c = scale was self-constructed by study authors; Q_b = heterogeneity between groups; *k* = number of effect sizes; *n* = combined sample size; *d* = average effect size (Random Effects); *95% CI* = 95% confidence interval of *d*; *p* = significance level of *d*; Q_w = heterogeneity statistic (within); *** $p < 0.001$

S10 - Scale type (established vs. self-constructed) as moderator of associations with mental health

Function of employment	*Scale type*	Q_b	*k*	*n*	*r*	*95% CI*	*p*	Q_w
TS * MH	est.	0.82	52	13730	0.22	0.21; 0.24	0.0000	479.08
	s-c		2	1309	0.25	0.19; 0.30	0.0000	10.79
SC * MH	est.	14.74***	44	12045	0.26	0.24; 0.28	0.0000	293.41
	s-c		15	6476	0.20	0.17; 0.22	0.0000	90.08
ST * MH	est.	15.62***	37	11940	0.30	0.28; 0.32	0.0000	448.70
	s-c		6	3939	0.37	0.34; 0.40	0.0000	25.31
AC * MH	est.	23.05***	36	10400	0.19	0.17; 0.21	0.0000	126.00
	s-c		12	4442	0.28	0.25; 0.31	0.0000	98.05
FS * MH	est.	1.24	47	15167	0.24	0.23; 0.26	0.0000	755.44
	s-c		21	11930	0.23	0.21; 0.25	0.0000	997.22

Scale type: est. = established scale, s-c = scale was self-constructed by study authors; MH = mental health; TS = time structure; SC = social contact; ST = status; AC = activity; FS = financial situation; Q_b = heterogeneity between groups; *k* = number of effect sizes; *n* = combined sample size; *r* = average correlation (Random Effects); *95% CI* = 95% confidence interval of *r*; *p* = significance level of *r*; Q_w = heterogeneity statistic (within); *** $p < 0.001$

S11 - Reference to deprivation model in article title as moderator of differences between employed and unemployed persons

Function of employment	*Reference to model*	Q_b	*k*	*n*	*d*	*95% CI*	*p*	Q_w
Combined LFs	no	25.14***	26	25877	0.53	0.50; 0.57	0.0000	897.74
	yes		10	3984	0.78	0.69; 0.87	0.0000	63.58
Time structure	no	3.37	9	2756	0.55	0.48; 0.63	0.0000	225.35
	yes		8	3540	0.67	0.58; 0.77	0.0000	77.38
Collect. Purpose	no	24.97	9	9032	0.36	0.31; 0.42	0.0000	259.14
	yes		7	3468	0.64	0.55; 0.74	0.0000	67.68
Social contact	no	42.18***	19	11944	0.35	0.31; 0.39	0.0000	197.35
	yes		8	3540	0.70	0.60; 0.79	0.0000	44.62
Status	no	26.23***	9	3365	0.39	0.31; 0.46	0.0000	1049.25
	yes		8	3540	0.63	0.54; 0.73	0.0000	74.80
Activity	no	112.44***	17	23583	0.45	0.42; 0.49	0.0000	476.83
	yes		7	3468	1.03	0.93; 1.13	0.0000	128.45
Manif. funct.	no	38.22***	12	14567	0.46	0.43; 0.50	0.0000	755.43
	yes		7	3477	0.81	0.71; 0.91	0.0000	128.36

Reference to model: Does the study title mention the deprivation model? Yes = model is mentioned, no = model is not mentioned; Combined latent functions = average of the effect sizes for each sample; Q_b = heterogeneity between groups; *k* = number of effect sizes; *n* = combined sample size; *d* = average effect size (Random Effects); *95% CI* = 95% confidence interval of *d*; *p* = significance level of *d*; Q_w = heterogeneity statistic (within); *** $p < 0.001$

S12 - Reference to deprivation model in article title as moderator of associations with mental health

Function of employment	*Reference to model*	Q_b	*k*	*n*	*r*	*95% CI*	*p*	Q_w
LF * MH	no	53.79***	37	9903	0.22	0.20; 0.24	0.0000	163.80
	yes		37	15347	0.31	0.30; 0.33	0.0000	379.68
TS * MH	no	7.71**	12	3010	0.25	0.22; 0.29	0.0000	134.56
	yes		26	10421	0.19	0.17; 0.21	0.0000	184.91
CP * MH	no	7.35**	7	3013	0.25	0.21; 0.28	0.0000	8.13
	yes		26	9708	0.30	0.29; 0.32	0.0000	331.97
SC * MH	no	16.97***	26	6009	0.17	0.15; 0.20	0.0000	90.15
	yes		28	10917	0.24	0.22; 0.26	0.0000	202.80
ST * MH	no	4.16*	13	3753	0.28	0.25; 0.31	0.0000	103.26
	yes		28	10917	0.32	0.30; 0.34	0.0000	367.62
AC * MH	no	14.02***	14	4065	0.29	0.26; 0.32	0.0000	70.53
	yes		27	10846	0.22	0.20; 0.24	0.0000	164.52
FS * MH	no	4.21*	35	13416	0.23	0.21; 0.25	0.0000	282.60
	yes		29	12181	0.20	0.19; 0.22	0.0000	1271.65

Reference to model: Does the study title mention the deprivation model? Yes = model is mentioned, no = model is not mentioned; MH = mental health; LF = combined latent functions; TS = time structure, CP = collective purpose; SC = social contact; ST = status; AC = activity; FS = financial situation; Q_b = heterogeneity between groups; *k* = number of effect sizes; *n* = combined sample size; *r* = average correlation (Random Effects); *95% CI*

= 95% confidence interval of r; p = significance level of r; Q_w = heterogeneity statistic (within); * $p < 0.05$, ** $p < 0.01$, *** $p < 0.001$

F) List of primary studies used in meta-analysis

** = study was used in the meta-analysis of group comparisons*

# = study was used in the meta-analysis of correlations between manifest/latent functions and mental health

Auslander, G. K. (1988). Social networks and health status of the unemployed. *Health & Social Work, 13*(3), 191-200.*

Banks, M. H. & Ullah, P. (1988). *Youth unemployment in the 1980s: Its psychological effects.* London: Croom Helm.#

Bjarnason, T. & Sigurdardottir, T. J. (2003). Psychological distress during unemployment and beyond: Social support and material deprivation among youth in six northern European countries. *Social Science & Medicine, 56*(5), 973-985.*#

Bolton, W. & Oatley, K. (1987). A longitudinal study of social support and depression in unemployed men. *Psychological Medicine, 17*(2), 453-460.*#

Bond, M. J. & Feather, N. T. (1988). Some correlates of structure and purpose in the use of time. *Journal of Personality and Social Psychology*, *55*(2), 321-329.#

Brief, A.P., Konovsky, M. A., Goodwin, R. & Link, K. (1995). Inferring the meaning of work from the effects of unemployment. *Journal of Applied Social Psychology, 25*(8), 693- 711.#

Bryce, J. & Haworth, J. (2003). Psychological well-being in a sample of male and female office workers. *Journal of Applied Social Psychology, 33*(3), 565-585.#

Brydsten, A., Hammarström, A. & San Sebastian, M. (2018). Health inequalities between employed and unemployed in northern Sweden: A decomposition analysis of social determinants for mental health. *International Journal for Equity in Health, 17*(1), 59.*

Congdon, D. C. (1990). Gender, employment and psychosocial well-being. *Journal of Sociology & Social Welfare, 17*(3), 101-121.*#

Creed, P. A. & Bartrum, D. A. (2008). Personal control as a mediator and moderator between life strains and psychological well-being in the unemployed. *Journal of Applied Social Psychology, 38*(2), 460-481.#

Creed, P. A. & Evans, B. M. (2002). Personality, well-being and deprivation theory. *Personality and Individual Differences, 33*(7), 1045-1054.#

Creed, P.A. & Klisch, J. (2005). Future outlook and financial strain: Testing the personal agency and latent deprivation models of unemployment and well-being. *Journal of Occupational Health Psychology, 10*(3), 251-260.#

Creed, P. A. & Machin, M. A. (2002). Access to the latent benefits of employment for unemployed and underemployed individuals. *Psychological Reports, 90*(3), 1208-1210.#

Creed, P. A. & Machin, M. A. (2003). Multidimensional properties of the access to categories of experience scale. *European Journal of Psychological Assessment, 19*(2), 85-91.#

Creed, P. A. & Macintyre, S. R. (2001). The relative effects of deprivation of the latent and manifest benefits of employment on the well-being of unemployed people. *Journal of Occupational Health Psychology, 6*(4), 324-331.#

Creed, P. A. & Muller, J. (2006). Psychological distress in the labour market: Shame or deprivation? *Australian Journal of Psychology, 58*(1), 31-39.*#

Creed, P. A. & Reynolds, J. (2001). Economic deprivation, experiential deprivation and social loneliness in unemployed and employed youth. *Journal of Community & Applied Social Psychology, 11*(3), 167-178.*#

Creed, P. A. & Watson, T. (2003). Age, gender, psychological well-being and the impact of losing the latent and manifest benefits of employment in unemployed people. *Australian Journal of Psychology, 55*(2), 95-103.#

Creed, P. A., Hicks, R. E. & Machin, M. A. (1998). Behavioural plasticity and mental health outcomes for long-term unemployed attending occupational training programmes. *Journal of Occupational and Organizational Psychology, 71*(2), 171-191.#

Creed, P. A., Muller, J. & Machin, M. A. (2001). The role of satisfaction with occupational status, neuroticism, financial strain and categories of experience in predicting mental health in the unemployed. *Personality and Individual Differences, 30*(3), 435-447.#

Creed, P. A., Muller, J. & Patton, W. (2003). Leaving high school: The influence and consequences for psychological well-being and career-related confidence. *Journal of Adolescence, 26*(3), 295-311.*#

Evans, S. T. & Banks, M. H. (1992). Latent functions of employment: variations according to employment status and labour market. In C. H. A Verhaar, L. G. Jansma, M. P. M. de Goede, J. A. C. van Ophem & A. de Vries (Eds.), *On the mysteries of unemployment. Causes, consequences and policies* (pp. 281-295). Dordrecht: Springer Science + Business Media.*#

Evans, S. T. & Haworth, J. T. (1991). Variations in personal activity, access to 'categories of experience', and psychological well-being in young adults. *Leisure Studies, 10*(3), 249- 264.*#

Feather, N. T. (1989). Reported changes in behaviour after job loss in a sample of older unemployed men. *Australian Journal of Psychology, 41*(2), 175-185.#

Feather, N. T. & Bond, M. J. (1983). Time structure and purposeful activity among employed and unemployed university graduates. *Journal of Occupational Psychology, 56*(3), 241- 254.*#

Frasquilho, D., Gaspar de Matos, M., Marques, A., Gaspar, T. & Caldas de Almeida, J. M. (2016). Distress and unemployment: The related economic and noneconomic factors in a sample of unemployed adults. *International Journal of Public Health, 61*(7), 821-828.#

George, J. M. (1991). Time structure and purpose as a mediator of work-life linkages. *Journal of Applied Social Psychology, 21*(4), 296-314.#

Gnambs, T., Stiglbauer, B. & Selenko, E. (2015). Psychological effects of (non)employment: A cross-national comparison of the United States and Japan. *Scandinavian Journal of Psychology, 56*(6), 659-669.#

Goodman, W. K., Geiger, A. M. & Wolf, J. M. (2015). Differential links between leisure activities and depressive symptoms in unemployed individuals. *Journal of Clinical Psychology, 72*(1), 70-78.*#

Goodman, W. K., Geiger, A. M. & Wolf, J. M. (2016). Leisure activities are linked to mental health benefits by providing time structure: comparing employed, unemployed and homemakers. *Journal of Epidemiology and Community Health, 71*(1), 4-11.*#

Hammer, T. (1993). Unemployment and mental health among young people: A longitudinal study. *Journal of Adolescence, 16*(4), 407-420.*#

Haworth, J. T. & Ducker, J. (1991). Psychological well-being and access to 'categories of experience' in unemployed young adults. *Leisure Studies, 10*(3), 265-274.#

Haworth, J. T., Jarman, M. & Lee, S. (1997). Positive psychological states in the daily life of a sample of working women. *Journal of Applied Social Psychology, 27*(4), 345-370.#

Haworth, J. T. & Millar, T. (1986). Time diary sampling of daily activity and intrinsic motivation in unemployed young adults. *Leisure Studies, 5*(3), 353-359.#

Haworth, J. T. & Paterson, F. (1995). Access to categories of experience and mental health in a sample of managers. *Journal of Applied Social Psychology, 25*(8), 712-724.#

Hayes, R. L. & Halford, W. K. (1996). Time use of unemployed and employed single male schizophrenia subjects. *Schizophrenia Bulletin, 22*(4), 659-669.*

Henwood, F. & Miles, I. (1987). The experience of unemployment and the sexual division of labour. In D. Fryer & P. Ullah (Eds.), *Unemployed people* (pp. 94-110). Milton Keynes: Open University Press.*

Hepworth, S. J. (1980). Moderating factors of the psychological impact of unemployment. *Journal of Occupational Psychology, 53*(2), 139-145.#

Hoare, P. N. & Machin, M. A. (2006). Maintaining wellbeing during unemployment. *Australian Journal of Career Development, 15*(1), 19-27.*#

Hultman, B. & Hemlin, S. (2008). Self-rated quality of life among the young unemployed and the young in work in northern Sweden. *Work, 30*(4), 461-472.*

Hultman, B., Hemlin, S. & Hörnquist, J. O. (2006). Quality of life among unemployed and employed people in northern Sweden. Are there any differences? *Work, 26*(1), 47-56.*

Isaksson, K. (1989). Unemployment, mental health and the psychological functions of work in male welfare clients in Stockholm. *Scandinavian Journal of Social Medicine, 17*(2), 165- 169.*#

Jackson, T. (1999). Differences in psychological experiences of employed, unemployed, and student samples of young adults. *The Journal of Psychology, 133*(1), 49-60.*#

Jackson, T., Iezzi, A. & Lafreniere, K. (1996). The differential effects of employment status on chronic pain and healthy comparison groups. *International Journal of Behavioral Medicine, 3*(4), 354-369.*#

Janlert, U. & Hammarström, A. (2009). Which theory is best? Explanatory models of the relationship between unemployment and health. *BioMed Central Public Health, 9*(1), 235.#

Johnson, A. M. & Jackson, P. M. (2012). Golden parachutes: Changing the experience of unemployment for managers. *Journal of Vocational Behavior, 80*(2), 474-485.#

Joshi, P., Garon, L. & Lechasseur, S. (1984). Self-esteem and loneliness among unemployed women. *Psychological Reports, 54*(3), 903-906.*#

Kelly, W. E. (2003). No time to worry: The relationship between worry, time structure, and time management. *Personality and Individual Differences, 35*(5), 1119-1126.#

Kokko, K. & Pulkkinen, L. (1998). Unemployment and psychological distress: Mediator effects. *Journal of Adult Development, 5*(4), 205-217.*#

Mallinckrodt, B. & Fretz, B. R. (1988). Social support and the impact of job loss on older professionals. *Journal of Counseling Psychology, 35*(3), 281-286.#

Martella, D. & Maass, A. (2000). Unemployment and life satisfaction: The moderating role

of time structure and collectivism. *Journal of Applied Psychology, 30*(5), 1095-1108.*#

Miles, I. (1983). *Adaption to unemployment?* SPRU Occasional Paper Series 20. Brighton: University of Sussex, Science Policy Research Unit.*#

Muller, J. J., Creed, P. M., Waters, L. E. & Machin, M. A. (2005). The development and preliminary testing of a scale to measure the latent and manifest benefits of employment. *European Journal of Psychological Assessment, 21*(3), 191-198.#

Paul, K. I. & Batinic, B. (2010). The need for work: Jahoda's latent functions of employment in a representative sample of the German population. *Journal of Organizational Behavior*, *31*(1), 45-64. *#

Paul, K. I., Geithner, E. & Moser, K. (2009). Latent deprivation among people who are employed, unemployed, or out of the labor force. *The Journal of Psychology, 143*(5), 477-491.*#

Paul, K. I. & Norvile, N. (2019). *Latent deprivation: Why depression is the main characteristic of unemployment-related distress*. Unpublished manuscript, University of Erlangen-Nuremberg. Available at: https://osf.io/6urmc/?view_only=d79e648a0c5049c393b3ec15279fa07e *

Price, R. H., Choi, J. N. & Vinokur, A. D. (2002). Links in the chain of adversity following job loss: How financial strain and loss of personal control lead to depression, impaired functioning, and poor health. *Journal of Occupational Health Psychology, 7*(4), 302-312.#

Rantakeisu, U., Starrin, B. & Hagquist, C. (1999). Financial hardship and shame: A tentative model to understand the social and health effects of unemployment. *The British Journal of Social Work, 29*(6), 877-901.#

Read, J. M., Muller, J. J. & Waters, L. E. (2013). The importance of latent benefits and meaningful leisure activity in predicting quality of life in Australian retirees. *Australian Journal of Career Development, 22*(2), 63-71.#

Reynolds, S. & Gilbert, P. (1991). Psychological impact of unemployment: Interactive effects of vulnerability and protective factors on depression. *Journal of Counseling Psychology, 39*(1), 76-84.#

Roberts, K., Lamb, K. L., Dench, S. & Brodie, D. A. (1989). Leisure patterns, health status and employment status. *Leisure Studies*, *8*(3), 229-235.*

Röhrle, B. & Hellmann, I. (1989). Characteristics of social networks and social support among long-term and short-term unemployed teachers. *Journal of Social and Personal Relationships, 6*(4), 463-473.#

Rowley, K. M. & Feather, N. T. (1987). The impact of unemployment in relation to age and length of unemployment. *Journal of Occupational Psychology, 60*(4), 323-332.#

Scanlan, J. N., Bundy, A. C. & Matthews, L. R. (2010). Investigating the relationship between meaningful time use and health in 18- to 25-year-old unemployed people in New South Wales, Australia. *Journal of Community & Applied Social Psychology, 20*(3), 232-247. #

Scanlan, J. N., Bundy, A. C. & Matthews, L. R. (2011). Promoting wellbeing in young unemployed adults: The importance of identifying meaningful patterns of time use. *Australian Occupational Therapy Journal, 58*(2), 111-119.*#

Selenko, E. & Batinic, B. (2013). Job insecurity and the benefits of work. *European Journal*

of Work and Organizational Psychology, 22(6), 725-736.#

Selenko, E. & Batinic, B. (2015). Volunteering as an alternative source of the benefits of work? Testing Jahoda's model among volunteer workers. Unpublished manuscript, Johannes Kepler University Linz.*#

Selenko, E., Batinic, B. & Paul, K. I. (2011). Does latent deprivation lead to psychological distress? Investigating Jahoda's model in a four-wave study. *Journal of Occupational and Organizational Psychology, 84*(4), 723-740.*#

Seršić, D. M. (2006). When does unemployment imply impaired psychological health? The mediating role of psychological deprivation and social support. *Review of Psychology, 13*(1), 43-50.#

Shamir, B. (1986). Unemployment and nonwork activities among persons with higher education. *The Journal of Applied Behavioral Science, 22*(4), 459-475.*#

Shams, M. (1993). Social support and psychological well-being among unemployed British Asian men. *Social Behavior and Personality, 21*(3), 175-186.#

Slack, K. J. (2004). *Examining job insecurity and well-being in the context of the role of employment*. Unpublished dissertation, University of Houston.#

Sousa-Ribeiro, M., Sverke, M. & Coimbra, J. L. (2014). Perceived quality of the psychosocial environment and well-being in employed and unemployed older adults: The importance of latent benefits and environmental vitamins. *Economic and Industrial Democracy, 35*(4), 629-652.#

Šverko, B., Galić, Z., Seršić, D. M. & Galešić, M. (2008). Unemployed people in search of a job: Reconsidering the role of search behavior. *Journal of Vocational Behavior, 72*(3), 415-428.#

Takahashi, M. & Winefield, A. H. (2014). Mental health of the unemployed in Japan. In M. F. Dollard, A. Shimazu, R. Bin Nordin, P. Brough & M. R. Tuckey (Eds.), *Psychosocial factors at work in the Asia pacific* (pp. 231-251). New York: Springer Science + Business Media.*

Tosti-Kharas, J. (2012). Continued organizational identification following involuntary job loss. *Journal of Managerial Psychology, 27*(8), 829-847.#

Tuncay, T. & Yildirim, B. (2015). Factors affecting the psychological distress among unemployed and re-employed individuals. *Career Development International*, *20*(5), 482-502.*

Underlid, K. (1996). Activity during unemployment and mental health. *Scandinavian Journal of Psychology, 37*(3), 269-281.#

van der Elst, T., Näswall, K., Bernhard-Oettel, C., De Witte, H. & Sverke, M. (2016). The effect of job insecurity on employee health complaints: A within-person analysis of the explanatory role of threats to the manifest and latent benefits of work. *Journal of Occupational Health Psychology, 21*(1), 65-76.#

Van Hoye, G. & Lootens, H. (2013). Coping with unemployment: Personality, role demands, and time structure. *Journal of Vocational Behavior, 82*(2), 85-95.#

Van Raaji, W. F. & Antonides, G. (1991). Costs and benefits of unemployment and employment. *Journal of Economic Psychology, 12*(4), 667-687.*

Viinamäki, H., Koskela, K., Niskanen, L. & Arnkill, R. (1993). Social support in relation to mental well-being among the unemployed: A factory closing study. *Nordic Journal of*

Psychiatry, 47(3), 195-201.#

Wahl, I., Pollai, M. & Kirchler, E. (2013). Status, identification and in-group favouritism of the unemployed compared to other social categories. *The Journal of Socio-Economics, 43*, 37-43.*

Walter-Tittmann, S. (2008). *Arbeitslosigkeit, Inkongruenz und psychische Gesundheit - Erwerbslose und Berufstätige im Vergleich.* Unveröffentlichte Diplomarbeit, Universität Erlangen-Nürnberg.*

Wanberg, C. R., Griffiths, R. F. & Gavin, M. B. (1997). Time structure and unemployment: A longitudinal investigation. *Journal of Occupational and Organizational Psychology, 70*(1), 75-95.*#

Warr, P. B., Banks, M. H. & Ullah, P. (1985). The experience of unemployment among black and white urban teenagers. *British Journal of Psychology, 76*(1), 75-87.#

Warr, P. B., Butcher, V., Robertson, I. & Callinan, M. (2004). Older people's well-being as a function of employment, retirement, environmental characteristics and role preference. *British Journal of Psychology, 95*(3), 297-324.*#

Waters, L. E. & Moore, K. A. (2001). Coping with economic deprivation during unemployment. *Journal of Economic Psychology, 22*(4), 461-482.#

Waters, L. E. & Moore, K. A. (2002a). Reducing latent deprivation during unemployment: The role of meaningful leisure activity. *Journal of Occupational and Organizational Psychology, 75*(1), 15-32.*#

Waters, L. E. & Moore, K. A. (2002b). Self-esteem, appraisal and coping: A comparison of unemployed and re-employed people. *Journal of Organizational Behavior, 23*(5), 593-604.*#

Waters, L. E. & Muller, J. J. (2003). Money or time? Comparing the effects of time structure and financial deprivation on the psychological distress of unemployed adults. *Australian Journal of Psychology, 55*(3), 166-175.#

Whelan, C. T. (1992). The role of income, life-style deprivation and financial strain in mediating the impact of unemployment on psychological distress: Evidence from the Republic of Ireland. *Journal of Occupational and Organizational Psychology, 65*(4), 331-344.#

Winefield, A. H. & Tiggemann, M. (1985). Psychological correlates of employment and unemployment: Effects, predisposing factors, and sex differences. *Journal of Occupational Psychology, 58*(3), 229-242.*

Winefield, A. H. & Tiggemann, M. & Winefield, H. R. (1990). Factors moderating the psychological impact of unemployment at different ages. *Personality and Individual Differences, 11*(1), 45-52.*#

Winefield, A. H. & Tiggemann, M. & Winefield, H. R. (1992). Spare time use and psychological well-being in employed and unemployed young people. *Journal of Occupational and Organizational Psychology, 65*(4), 307-313.*#

Zechmann, A., & Paul, K. I. (2019). Why do individuals suffer during unemployment? Analyzing the role of deprived psychological needs in a six-wave longitudinal study. *Journal of Occupational Health Psychology, 24*(6), 641-661.
